# Unlocking artificial intelligence, machine learning and deep learning to combat therapeutic resistance in metastatic castration-resistant prostate cancer: a comprehensive review

**DOI:** 10.3332/ecancer.2025.1953

**Published:** 2025-07-29

**Authors:** Zainab Haider Ejaz, Reyan Hussain Shaikh, Alizeh Sonia Fatimi, Saqib Raza Khan

**Affiliations:** 1 Aga Khan University Hospital, Aga Khan Medical College, Sindh, Karachi 748000, Pakistan; 2 Division of Medical Oncology, Department of Oncology, Schulich School of Medicine & Dentistry, Western University, London, ON N6A 3K7, Canada; 3 Verspeeten Family Cancer Centre, London Health Sciences Centre, London, ON N6A 5W9, Canada; a https://orcid.org/0000-0002-3742-534X

**Keywords:** prostate cancer, castration resistance, metastasis, artificial intelligence, therapeutic resistance

## Abstract

Metastatic castration-resistant prostate cancer (mCRPC) remains a formidable clinical challenge despite advancements in therapy. This narrative review explores the role of artificial intelligence (AI), machine learning and deep learning in addressing therapeutic resistance in mCRPC. AI-driven approaches leverage integrated datasets encompassing genomics, proteomics and clinical parameters to uncover molecular mechanisms, predict treatment responses and identify biomarkers of resistance. These methodologies promise personalised treatment strategies tailored to individual patient profiles. However, data heterogeneity and regulatory considerations are challenges that hinder the translation of AI insights into clinical practice. By synthesising current literature, this review examines the progress, potential and limitations of AI applications in combating therapeutic resistance in mCRPC, highlighting implications for future research and clinical implementation.

## Background

Castration-resistant prostate cancer (CRPC) is defined by the progression of prostate cancer, whether detected radiologically or biochemically, despite undergoing standard androgen deprivation therapy (ADT), where serum testosterone levels have decreased to castration levels (usually below 50 ng/dl or 1.7 nmol/l) [1]. Metastatic castration-resistant prostate cancer (mCRPC) is an aggressive form of cancer, prevalent among men and is associated with elevated morbidity and mortality [2]. With millions affected globally, mCRPC presents a formidable clinical challenge due to its aggressive nature and tendency to develop resistance to treatment. Despite advancements in oncological research and therapies, managing mCRPC remains difficult, as traditional approaches frequently fail to effectively halt disease progression or enhance patient outcomes [3].

The emergence of resistance mechanisms against frontline therapies, such as ADT and oral hormonal agents like enzalutamide and abiraterone underscore the urgent need for innovative strategies to address therapeutic resistance in mCRPC [4, 5]. The need for more effective interventions to combat this disease has spurred a growing interest in harnessing the power of artificial intelligence (AI), machine learning (ML) and deep learning (DL) to decipher its underlying complexities and devise personalised treatment regimens tailored to individual patient profiles [6–8].

In recent years, the convergence of AI, ML and DL technologies has propelled significant breakthroughs across various domains of healthcare, offering unprecedented opportunities for precision medicine, early disease detection and treatment optimisation [9]. Within the realm of mCRPC, the application of computational methodologies holds immense promise in elucidating the intricate molecular pathways driving therapeutic resistance and identifying novel targets for intervention. By analysing vast datasets encompassing genomics, proteomics and clinical parameters, AI-driven approaches can elucidate the molecular basis of treatment or disease progression or aid in the discovery of predictive biomarkers of the disease [10, 11].

This comprehensive review endeavors to explore the current landscape of AI-driven solutions aimed at overcoming therapeutic resistance in mCRPC.

## Overview of artificial intelligence, machine learning and deep learning

In recent years, AI, ML and DL have garnered significant attention in oncology due to their potential to revolutionise research and clinical practice [12–14]. AI refers to the utilisation of a variety of techniques to enable computers to carry out simulated tasks requiring human intelligence [15].

ML, a subset of AI, involves the development of algorithms where computers learn the data and make predictions or decisions without explicit instructions [16, 17]. ML has been used in cancer research to analyse intricate data relationships and make predictions about cancer outcomes. Many studies have explored the utility of ML in cancer research and have highlighted its potential and limitations. Decision trees are a specific ML technique that has demonstrated broad applicability across various cancer types, significantly improving diagnostic accuracy [18–20]. These applications encompass various cancer types, including breast, gastric, thyroid, prostate and colorectal cancer. Additionally, other ML algorithms such as k-means, K-nearest neighbors [21], logistic regression [22], Naïve Bayes [23, 24], principal component analysis [25, 26], Random Forests [27, 28, 29] and eXtreme Gradient Boost [30] have also been employed in cancer research, contributing to improved diagnostic accuracy and prediction across various cancer types [31].

DL is a specialised form of ML where multilayered artificial neural networks are employed to extract complex features from vast datasets, enabling the creation of powerful models that are able to capture complicated patterns [32]. DL has emerged as a transformative technology in cancer research. It can learn intricate features from expansive datasets automatically, making it a powerful tool for analysing images and molecular profiling. Studies that have explored the application of DL have shown its remarkable potential in improving diagnosis, predicting disease progression and exploring new treatment options [33, 34]. Moreover, initiatives such as the introduction of expansive pathological image datasets, such as SNOW for breast cancer research, have significantly improved computational pathology by providing abundant data for training robust DL models [35]. Furthermore, the development of DL-based methods for the automatic identification of circulating tumour cells and cancer-associated fibroblasts has shown superior performance compared to conventional computer vision methods [35]. 

The widespread success of AI, ML and DL in improving diagnostics and treatment strategies across various cancer types underscores their potential utility in addressing the complexities of mCRPC. Evidence from studies in breast, gastric and colorectal cancer highlights the significant contributions of these technologies in enhancing diagnostic accuracy and predicting therapeutic responses. Utilising the capabilities of AI, ML and DL in mCRPC research and clinical practice could lead to personalised therapeutic interventions tailored to individual patient profiles, ultimately improving outcomes for those affected by this aggressive form of prostate cancer. Thus, the demonstrated efficacy of AI-driven approaches in other cancer types suggests a promising avenue for combating therapeutic resistance in mCRPC through interdisciplinary collaboration and innovative application of these methodologies.

## Pathogenesis of metastatic castration-resistant prostate cancer

Patients diagnosed with localised prostate cancer usually undergo radical prostatectomy and/or radiotherapy, followed by ADT [36, 37]. Based on the cancer grade, a varying percentage of these patients progress to CRPC by one decade [38, 39]. CRPC, previously known as ‘hormone-refractory prostate cancer’ and ‘androgen-independent prostate cancer,’ still relies on hormone activity for androgen receptor (AR) activation, even though castration treatments like ADT were ineffective [40–42]. Consequently, the terms ‘hormone-refractory prostate cancer’ and ‘androgen-independent prostate cancer’ were replaced with ‘castration-resistant prostate cancer (CRPC)’ [43, 44].

It is widely recognised that patients with metastatic prostate cancer undergoing ADT tend to show disease progression after an average of 18–36 months [45]. The stem cell model of the prostate highlights that hormone resistance can arise through multiple pathways [46]. 

The normal prostate is maintained by a small number of androgen-independent stem cells that continually replenish themselves and produce androgen-sensitive cells [47]. When testosterone levels are within the physiological range, these androgen-sensitive cells differentiate into glandular epithelial cells until the cell population reaches equilibrium, balancing proliferation and cell death to prevent excessive growth [47]. As a result, the typical prostate consists predominantly of androgen-dependent glandular cells, a smaller number of androgen-sensitive basal cells and a limited population of androgen-independent basal stem cells [47].

Although the precise mechanisms are not entirely clear, several factors likely contribute to androgen independence, including genetic instability due to alterations in microenvironmental, impaired detoxification, activation of oncogenes and increased levels of androgen receptors [47]. 

## Mechanisms of therapeutic resistance in mCRPC

The therapeutic landscape in mCRPC is predominantly limited to taxane-based chemotherapies, with emerging secondary regimens such as cabazitaxel and AR-targeted therapies like abiraterone, enzalutamide and apalutamide [43, 48]. However, resistance to these therapies poses a significant challenge, driving ongoing research into new targets and drug development strategies for mCRPC.

Resistance mechanisms in mCRPC are intricate and diverse. Understanding these mechanisms is crucial for devising effective therapeutic strategies. A primary mechanism involves the overexpression of efflux proteins such as P-glycoprotein and multidrug resistance proteins, which expel the chemotherapy drugs from tumour cells, decreasing their effectiveness [43, 49].

The AR signalling pathway is involved in mCRPC progression, and resistance mechanisms against AR-targeted medications like abiraterone and enzalutamide are well-documented. Mutations in the ligand-binding domain (LBD) of AR can alter its conformation, causing receptor antagonists to act as agonists, thereby promoting resistance [49]. Up to 64% of mCRPC patients exhibit AR overexpression or amplification, while AR ovlike AR-V7, lacking the LBD, remain constitutively active and contribute further to therapeutic resistance [50, 51]. AR overexpression could be due to overexpression of AR coregulators, increased stability of the receptor itself or gene amplification resulting in increased phosphorylation or acetylation of histones at AR enhancers [52, 53]. Additionally, glucocorticoid receptor (GR) upregulation has been implicated in enzalutamide resistance, as both AR and GR share a binding site on the chromosome, leading to the activation of AR-specific genes [54, 55].

Another critical resistance mechanism involves tumour cells evading destruction by inhibiting apoptosis and repairing DNA damage, processes regulated by non-coding RNAs that modulate gene expression [56] Epigenetic changes also play a key role in mCRPC, such as covalent modifications of histone proteins, which ultimately affect the normal processes associated with DNA, thereby playing a role in disease progression [57].

Neuroendocrine differentiation of tumour cells promotes lineage plasticity, whereby cells show increased adaptability to survival by adapting to their environment [58, 59]. This concept of plasticity has also been suggested as a possible mechanism of therapeutic resistance and disease progression [60].

Activation of PI3K and AKT in mCRPC offers alternative survival pathways for the tumour cells, promoting cell growth and multiplication [61, 62]. The prostate tumour microenvironment is another contributing factor, involving a complex interaction between tumour cells, stromal cells and various immune components [63]. This environment is highly immunosuppressive, driven by inflammatory signals and low oxygen levels, which create a protective barrier around cancer cells [63]. Immune-suppressive cells, such as regulatory T cells, M2 macrophages and myeloid-derived suppressor cells, dominate the microenvironment, impeding the anti-tumour activities of crucial immune cells like dendritic cells, natural killer cells, B cells and cytotoxic T cells [63]. This suppression ultimately leads to resistance against treatments.

Poly-ADP ribose polymerase (PARP) inhibitors may also be used in treating mCRPC, but resistance mechanisms including aberrations in genes of homologous recombination repair (HRR) such as BRCA1, BRCA2 and PALB2, pose significant challenges [64]. Efflux transporters like ABC transporters also reduce intracellular concentrations of PARP inhibitors, contributing to resistance [65].

Despite early success, resistance to ^177^ Lu-PSMA radioligand therapy in mCRPC arises from its low linear energy transfer, causing primarily single-strand DNA breaks. In addition, the effectiveness of this therapy depends on PSMA-mediated uptake of the radioligand; tumours with low or heterogeneous PSMA expression may not receive adequate radiation doses, leading to reduced therapeutic efficacy and prostate-specific antigen (PSA) response [66]. 

**Figure 1.**
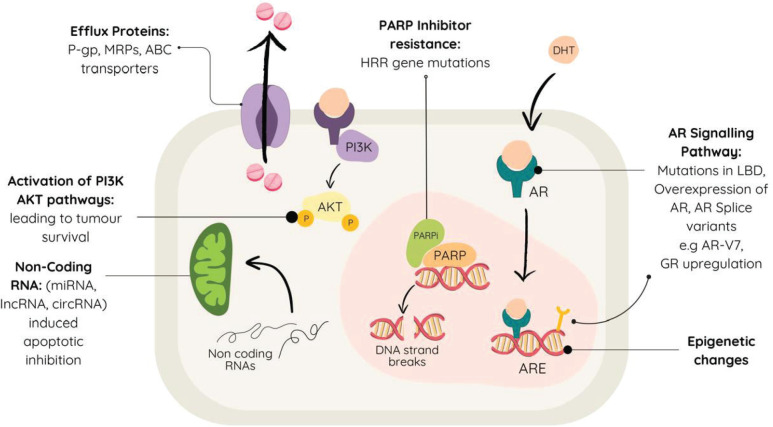
Various methods of therapeutic resistance in metastatic castration-resistant prostate cancer.

These examples, summarised in Figure 1, underscore the complexity of resistance mechanisms in mCRPC, highlighting the need for innovative approaches to overcome them. 

## Current standard of care in the treatment of metastatic castration-resistant prostate cancer

The National Comprehensive Cancer Network (NCCN) guidelines offer a multifaceted approach to managing mCRPC, advocating for the continuation of ADT alongside oral hormonal agents, chemotherapies such as docetaxel and oral targeted agents including abiraterone and enzalutamide, which are preferred choices if not previously administered [67].

Clinical decision-making in mCRPC hinges on various factors including prior treatments, patient preferences, symptomatology, adverse effects and the presence of visceral disease [67]. Regular monitoring via radiographic imaging (e.g., Computerised Tomography (CT) scans, bone imaging, prostate-specific membrane antigen positron emission tomography (PSMA PET) scans), clinical examinations and laboratory tests (e.g., PSA) are essential [67]. Therapy is typically continued until disease progression or intolerable side effects develop, as per NCCN guidelines [67].

Numerous novel therapies have been tested in clinical trials, summarised in Table 1, offering potential avenues for mCRPC treatment. Despite their demonstrated efficacy, overall survival rates remain low, highlighting the ongoing necessity for extensive clinical trials to establish definitive outcomes and for the developing new drugs and therapies to combat this problem.

## Applications of artificial intelligence, machine learning and deep learning in combating therapeutic resistance

**Figure 2.**
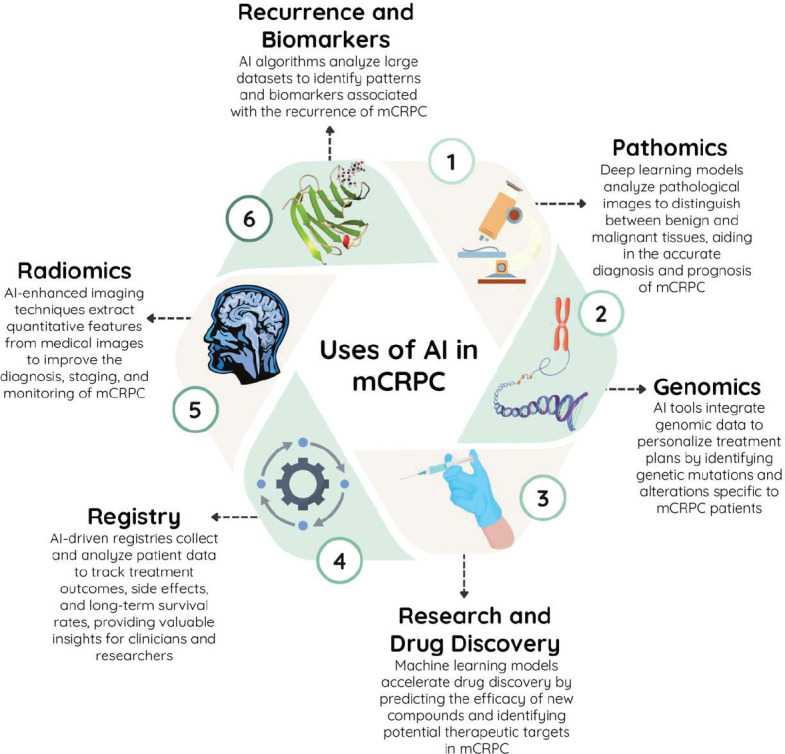
Different applications of artificial intelligence in the treatment of metastatic castration-resistant prostate cancer.

AI holds immense potential to transform the landscape of mCRPC treatment by revolutionising various aspects of patient care. Figure 2 illustrates the diverse applications of AI in targeting mCRPC. AI algorithms can analyse patient data, including genomic profiles, imaging results and treatment histories, to predict individual responses to various treatment modalities. By identifying individual patient characteristics, clinicians can create customised treatment plans that optimise therapeutic benefits while reducing adverse effects.

Table 2 summarises the current literature on AI applications in the diagnosis and treatment of prostate cancer.

### Pathomics

While prostate biopsy and Gleason scoring have historically served as the cornerstone for diagnosing localised prostate cancer, they are typically performed early in the disease course and often precede the development of mCRPC by several years [93]. Although prostate biopsy is not routinely required for the diagnosis of mCRPC, it retains a critical role in select clinical scenarios, particularly for molecular profiling and therapeutic stratification. Biopsies of metastatic sites are increasingly employed to enable mutational analysis and immunohistochemistry, identifying actionable genomic alterations such as BRCA1/2, ATM and MSI-H, which can guide personalised treatment decisions [94]. Moreover, tissue biopsy plays a role in ruling out neuroendocrine or small cell transformation, which warrants a distinct therapeutic approach [95]. Despite 58% of patients in one study being found to harbor theoretically actionable mutations, only a small subset received matched therapies, highlighting the disparity between molecular findings and clinical application [94]. Beyond traditional histopathology, AI has demonstrated considerable promise in enhancing pathology workflows. In a meta-analysis by Morozov *et al* [93], AI-assisted histological assessment across 8,000 prostate biopsies and 458 prostatectomy cases achieved diagnostic accuracies ranging from 83.7% to 98.3%. Similarly, the PANDA challenge led by Bulten *et al* [96], utilising over 10,000 digitised biopsies, showcased AI’s potential to improve the reproducibility and efficiency of Gleason grading. 

Jung *et al* [97] validated DeepDx^®^ Prostate (DeepDx), an AI-based diagnostic tool, using 593 whole-slide images (130 normal, 463 adenocarcinomas) against Gleason scores and grade groups assessed by three expert uropathologists as the reference standard [97]. DeepDx demonstrated comparable cancer detection accuracy to original pathology reports but showed higher concordance with reference grade groups and Gleason scores. In another study, Paige Prostate, a clinical-grade AI tool, was evaluated for its efficacy in aiding pathologists with identification, grading and quantifying prostate cancer in 105 prostate core needle biopsies (CNBs) [100]. Four pathologists initially diagnosed the CNBs independently, achieving a diagnostic accuracy of 95.0%.

**Figure 3.**
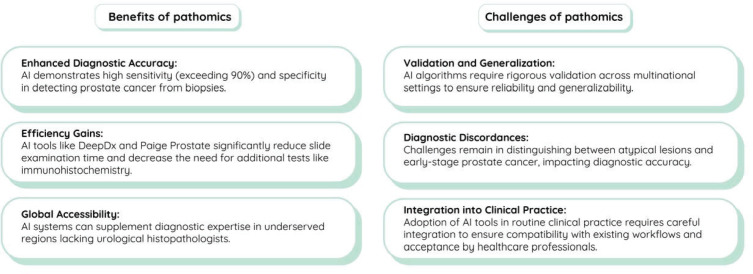
Benefits and challenges of pathomics in the diagnosis of metastatic castration-resistant prostate cancer.

While these advancements have primarily focused on localised disease, their integration into molecular pathology pipelines and application to metastatic lesion evaluation remain promising avenues for optimising precision oncology in mCRPC. In addition, early detection of prostate cancer allows for timely intervention, significantly impacting mCRPC prognosis. Detecting prostate cancer early increases the likelihood of successful localised treatment with surgery or radiation therapy, potentially preventing or delaying progression to advanced stages, including mCRPC. This timely intervention facilitates the early initiation of systemic therapies and enables more effective disease monitoring and management. Figure 3 summarises the benefits and potential challenges of incorporating AI into pathomics related to mCRPC diagnosis.

### Radiomics

The field of radiomics, which involves developing computational systems to extract and analyse quantitative features from medical images, holds promise for timely metastasis detection in mCRPC.

Wang *et al* [99] investigated the utility of texture features taken from multiparametric magnetic resonance imaging (mp-MRI) to predict bone metastases in prostate cancer patients [99]. By analysing 976 features from T2-weighted (T2-w) and dynamic contrast-enhanced T1-weighted (DCE T1-w) MRI scans of 176 patients, they found that combining information from both MRI sequences showed improved prognostic performance compared to using either sequence alone. Another study by Li *et al* [100] focused on developing an AI model using transrectal ultrasonography (TRUS) images to predict prostate cancer, comparing its diagnostic performance to radiologists and a clinical model [100]. Using 1,696 2-dimensional TRUS images from 142 patients, they trained a ResNet50 network with three classification models: original image (Whole), biopsy needle tract (Needle) and combined image (Feature Pyramid Networks (FPN)). The FPN model outperformed others.

Faiella *et al* [101] examined the role of AI models in detecting and predicting lymph node involvement (LNI) in prostate cancer patients. They evaluated 16 studies, where AI models predicted LNI as accurately as traditional nomograms, while CT and PET-CT based models showed strong diagnostic and prognostic capabilities.

**Figure 4.**
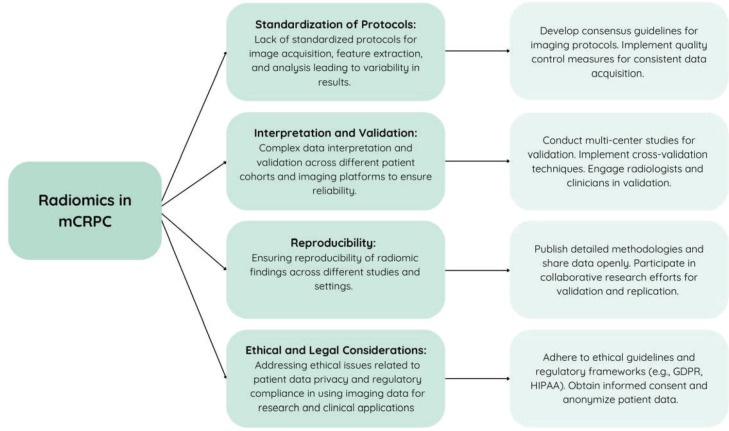
Challenges of incorporating radiomics in the treatment of metastatic castration-resistant prostate cancer and their solutions.

Radiomics' ability to analyse texture features from medical imaging offers early detection potential for BM in mCRPC, aiding in treatment planning and prognosis assessment. Its quantitative approach complements traditional imaging modalities, potentially enhancing sensitivity and specificity, thereby advancing precision medicine in mCRPC management. Figure 4 outlines anticipated challenges associated with radiomics in mCRPC and proposes strategies to overcome them.

### Genomics

Decipher, Oncotype DX Genomic Prostate Score and Prolaris are commercially available genomic tests, powered by ML, that guide prostate cancer management [102]. Decipher analyses gene expression to predict disease recurrence post-treatment. Oncotype DX Genomic Prostate Score assesses tumour aggressiveness to guide treatment decisions, especially post-surgery. Prolaris measures cell proliferation genes to predict disease aggressiveness and guide treatment strategies. These classifiers enable personalised treatment plans, sparing low-risk patients from unnecessary interventions while identifying high-risk cases needing more aggressive therapy. By integrating genomic data with clinical parameters, they enhance precision medicine, improving patient outcomes and quality of life. Clinicians can utilise these tools to tailor treatments, reducing overtreatment and minimising side effects. Their adoption signifies a shift towards data-driven decision-making in oncology, optimising patient care by harnessing the power of ML and genomics. 

Dadhania *et al* [103] used digitised slides of hematoxylin and eosin staining and identified the rearrangement of TMPRSS2-ERG in prostate cancer using a DL algorithm. Image patches from slides from 392 cases were exported at various magnifications. A DL model based on MobileNetV2 architecture was trained separately for each magnification level. The area under the curve (AUC) of the receiver operating characteristic (ROC) curves ranged between 0.82 and 0.85. The sensitivity of the models was 75.0% and the specificity was 83.1% (20 × model). Mena *et al* [104] developed an ML-based classifier using 47 genes selected for their differential expression in prostate cancer, gene ontology and supporting literature. This classifier was trained using data available from 550 samples obtained from 'The Cancer Genome Atlas' and subsequently validated on four diverse external datasets comprising a total of 463 samples. Strong statistical significance was shown for the most successful strategy, which combined the Random Forest method with majority class downsampling. Across all datasets, their approach consistently produced an average sensitivity of 0.90, specificity of 0.80 and AUC of 0.84.

Genomics offers a promising opportunity for personalised treatment strategies. By identifying specific gene mutations or rearrangements, the most ideal drug therapy can be suggested and its efficacy can be predicted beforehand. For instance, genomic profiling can reveal mutations in DNA repair genes like BRCA1/2 or alterations in the AR signalling pathway, which can guide the use of targeted therapies such as PARP inhibitors for BRCA-mutated cancers or next-generation AR signalling inhibitors. Moreover, clinical trials can be enhanced by stratifying the participants on genomic data. By focusing on genomic-driven drug development, newer and more effective therapies can emerge for mCRPC.

### Recurrence prediction

Huang *et al* [105] presented a novel AI-powered method for predicting an early recurrence of prostate cancer post-prostatectomy and identifying cancer driver regions. Using deep convolutional neural networks, the study developed an AI model trained on whole slide images and patient data. The model extracted both visual and microscopic morphological features to find predictive regions of early recurrence (regions of interest [ROIs]). The model demonstrated promising results, with AI-derived morphometric scores effectively ranking regions of ROIs and showing strong prognostic value for 3-year biochemical recurrence (AUC = 0.78), significantly outperforming the traditional Gleason Grade Group, which had an AUC of 0.62. 

Liu *et al* [106] in their systematic review evaluates the effectiveness of AI in predicting biochemical recurrence after prostate cancer surgery. AI algorithms, especially those using radiological features, demonstrated higher accuracy in predicting the recurrence compared to those based on pathological or clinicopathological data. In some cases, AI outperformed traditional prediction methods. However, the review also identified significant variability in AI performance due to differences in study designs, patient inclusion criteria and follow-up data.

Eminaga *et al* [107] developed an AI-based system to predict prostate cancer recurrence using histology images. Validated with multi-institutional datasets of 2,647 patients over 10 years, the system demonstrated superior predictive accuracy compared to existing grading systems. It categorised prostate cancer into four distinct risk groups and showed high consensus among experts, suggesting that AI can enhance recurrence prediction and improve clinical decision-making for patients.

These studies underscore the potential of AI to accurately predict the recurrence of prostate cancer.

### Biomarker discovery

In addition, Huang *et al* [105] identified a potential new prostate cancer biomarker, TMEM173, associated with the STING pathway, through focused biomarker analysis of high-scored ROIs. This research introduced an innovative approach to identifying prostate cancer patients at risk for early recurrence and discovering novel biomarkers, utilising AI to analyse morphologic features from whole slide image data.

### Research and drug discovery

CancerOmicsNet is a novel system based on AI that predicts how well the multitargeted kinase inhibitors will work against various types of cancer using a deep graph learning model [108]. Gagare *et al* [109] utilised ML and DL algorithms to train predictive models using molecular features, achieving high accuracy on both training and test datasets. It is important to further validate these models using experimental assays to establish their full reliability and utility.

Nevertheless, AI algorithms can analyse large-scale biomedical data, including genomic data and clinical trial results, to identify potential biomarkers, therapeutic targets and novel drug candidates for prostate cancer. This accelerates the process of drug development, allowing more effective therapies. 

### Registry management

Maintaining an efficient registry for metastatic prostate cancer is crucial for providing the latest data to clinicians. However, using electronic health records (EHRs) that are mainly present in the form of text, to manage patient data poses challenges. Recent advancements in AI technologies, such as named entity recognition and natural language processing (NLP) have enabled the acquisition of valuable information from large amounts of unstructured textual data (text mining) [110]. In the Netherlands, NLP-based text-mining software is widely used across hospitals to semiautomatically gather data from EHRs [110]. This registry enables clinicians and researchers to access updated data, analyse treatment trends, monitor disease progression and evaluate patient outcomes. Access to such data is critical for making informed treatment decisions, modifying strategies based on the latest outcomes and adapting to emerging trends in patient care.

## Ongoing trials incorporating artificial intelligence to develop robust algorithms in the management of prostate cancer

Currently, multiple trials are ongoing in oncology and AI (Table 3). By incorporating AI with diagnostic imaging, these trials hold immense potential for developing robust algorithms and models that can facilitate accurate and prompt diagnosis. Continuous follow-up of these trials can provide valuable insights into treatment responses and assist in predicting therapeutic outcomes based on AI's ability to analyse images. This integration of AI offers an opportunity to adjust therapeutic strategies, allowing for the most suitable treatment approach for the patient without compromising the quality of healthcare.

## Studies involving artificial intelligence to reduce therapeutic resistance in CRPC

Several studies have explored the integration of AI to understand therapeutic resistance and the effects of various therapies on a cellular level in CRPC. For instance, Blatti *et al* [10] developed a tool called TraRe, which used RNA sequencing to determine the response of abiraterone in mCRPC in different transcriptional networks. TraRe pinpointed a specific transcriptional module linked to the immune response, enriched with rewired regulatory networks in abiraterone treatment responders versus non-responders. This module included key transcription factors such as CEBPE, GATA1, KLF1 and MYB, known to influence granulocyte differentiation, erythroid development and various hematopoietic and immune pathways. Additionally, TAL1 and NFE2 emerged as pivotal factors in gene expression regulation. Furthermore, TraRe identified transcription factors like SREBF2, SMAD7, SOX8 and SNAI2 driving other rewired transcriptional modules potentially implicated in cancer progression and resistance mechanisms. The analysis also highlighted the crucial role of three transcription factors (ELK3, MYB and MXD1) which functioned differently in patients who didn't respond well to treatment. These findings underscore the complexity of transcriptional rewiring in mCRPC and the potential utility of TraRe in formulating personalised treatment plans.

Xue *et al* [117] predicted dosages using an ML technique for mCRPC patients. The patients’ PET imaging data and clinical information were used in the ML model as inputs, while Hermes software was utilised to calculate the organ-level dosimetry. Using standard uptake values, the ML algorithms predicted the dosimetry at the organ level. This algorithm can potentially improve treatment for CRPC by personalising radiation plans to maximise tumour kill while minimising damage to healthy organs. Through the prediction of individual radiation tolerance, this method may enable more aggressive dosing without compromising safety thresholds.

AI models can also be used to predict treatment discontinuity due to adverse effects. An example of this would be the algorithm laid out by Deng *et al* [118] which integrated data from three cohorts from different clinical trials. Various ML models were tested, including linear regression, cox regression, logistic regression and nonlinear tree-based methods. Tree-based models performed better, with random forest (RF) showing the highest performance in two out of the three cohorts. This RF model was then trained to identify patterns that differentiate patients who discontinue treatment from those who continue, with certain data features, such as laboratory test results and medical history, having more influence on the model’s predictions. This model could help personalise treatment plans for CRPC, potentially reducing unwanted side effects and improving adherence and tolerance to treatment.

AI also has the ability to identify genomic mutations in mCRPC, which can then be used to identify different targets for drug development. One such study was conducted by Lin *et al* [119] which explored the next-generation sequencing of circulating cell-free DNA and used ML algorithms to identify specific genomic alterations to distinguish patients with castration-resistant from those with castration-sensitive prostate cancer (CSPC). This study revealed potential genomic alterations in patients with mCRPC, specifically in the PI3K, RTK, G1/S and MAPK signalling pathways, highlighting the need to potentially target these pathways for therapeutic purposes.

## Challenges and limitations of successful implementation of artificial intelligence to combat therapeutic resistance in castration-resistant prostate cancer

The successful implementation of AI in healthcare, particularly in combating therapeutic resistance in CRPC, faces numerous challenges. Firstly, ML and DL algorithms rely on vast datasets, which often present problems since hospital data is considered confidential and not commonly shared between institutions [120, 121]. This challenge of data accessibility hinders the development of complex algorithms, especially because ML-based systems depend on continuous improvement through training with expanding datasets [122]. The large data required also necessitates the establishment of an AI-supporting platform that can hold large amounts of data for storage, processing and analysis [123]. This is critical to understanding and predicting therapeutic resistance patterns effectively. Unfortunately, establishing such platforms involves expensive hardware, software and skilled personnel, costs that are often too high for individual research teams to afford [123].

Heterogeneity in disease presentation and progression presents a special difficulty in the setting of prostate cancer, especially in mCRPC, which involves a variety of genetic subtypes (e.g., BRCA1/2-mutated, AR-V7 positive, neuroendocrine transformation). The generalisability of the model may be hampered by this variability. For example, when applied to mCRPC populations, where therapeutic resistance mechanisms differ dramatically from hormone-sensitive illness, AI models trained on localised prostate cancer datasets may perform poorly. Another limitation is data labeling and annotation complexity, especially in histopathology. While AI has demonstrated promise in tasks such as Gleason grading, training models to detect subtle features like neuroendocrine differentiation or intratumoural heterogeneity - hallmarks of advanced mCRPC - is far more complex, often requiring expert manual annotation and large sample diversity.

Another potential issue concerns patient consent and data privacy. A prominent example occurred in 2018 with the acquisition of DeepMind Health by Google. Their application, Streams, was created to manage patients with acute kidney injuries. However, the project faced criticism when it was revealed that the National Health Service (NHS) had shared sensitive data of more than 1 million patients with DeepMind servers without obtaining patient consent [124]. This underscores the critical issue of patient consent, as healthcare data can be easily breached given its sensitivity [125]. The emergence of AI adds to these complications, as individuals may inadvertently grant access to further data collection by mistaking AI systems for human interaction [126]. Thus, it is crucial for AI to play a safe and ethical role in healthcare by adhering to medical ethics and laws. Additionally, specific regulations governing the development and implementation of such technologies are warranted [127]. In mCRPC, patient consent and privacy are paramount given the confidentiality of cancer data. 

AI algorithms are also not perfectly reliable, and uncorrected errors within AI systems can lead to negative outcomes that may have significant impacts [128]. This is exemplified by an AI application built to assess the risk of post-pneumonia complications, which malfunctioned and incorrectly recommended discharging asthmatic patients, posing a potential health risk [129]. In prostate cancer, incorrect interpretation of imaging or biopsy data could result in misclassification of disease stage or resistance profile, leading to suboptimal treatment selection. This raises a pertinent problem regarding accountability: who is responsible for the mishap when the AI system makes an error? With a lack of guidelines on the ethical use of AI/ML/DL algorithms in healthcare, there is ambiguity in their use in hospitals, leading to reluctance to implement these technologies [130].

As described by Laranjo *et al* [131] current research in the field of diagnosis using ML techniques often lacks consistency in reporting and fails to address the practical needs of end-users [131]. The current focus rests primarily on assessing technical performance using historical data, neglecting the crucial ‘last mile’ of clinical evaluations through randomised trials that assess real-world clinical use of AI [132, 133]. This creates a problem with a lack of replication of trials, limiting the evidential data and potentiating the risk of methodological error [134]. The capacity to independently validate research findings is the cornerstone of rigorous scientific investigation, which is accomplished by replication studies that effectively replicate the original experiment under controlled settings [131]. While there are currently limited replication studies in health informatics [135], the problem extends further with the fact that some replication studies are unable to produce the same results as the original study, creating problems regarding the clinical implementation of AI programs [136].

Another problem regarding AI technologies is the lack of transparency, classically described as the ‘black box problem’ [137, 138, 139]. DL algorithms often fail to provide justifications for their predictions, presenting legal difficulties as the system cannot justify potentially erroneous recommendations [140]. Lack of transparency also fails to establish patients’ trust in the system, posing problems regarding the practical implementation of these systems in clinics [141]. This is especially problematic in prostate cancer, where treatment decisions hinge on nuanced variables such as androgen receptor splice variants, genomic alterations and immunohistochemical profiles. Without clear justifications for AI outputs, clinicians may hesitate to trust these models, impeding clinical adoption. 

Data heterogeneity presents another problem. Clinical data might differ greatly in terms of quality and presentation. This is best exemplified by surgical notes that are often varied and heterogeneous [123]. These differences may make it difficult for AI models trained on certain datasets to generalise to new data sources. This is compounded in prostate cancer and CRPC by variations in reporting standards across biopsy pathology, MRI protocols and molecular testing platforms, making generalisation across centers difficult. This highlights the need to maintain datasets containing cleaned data with a maximal signal-to-noise ratio to ensure robustness and reliability for the widespread application of AI techniques [123].

Another significant reason for the reluctance to adopt AI is the belief that AI will replace the human workforce [121]. This is a flawed perspective in medicine as it is a dynamic field with unpredictable circumstances that often require intuition and innate abilities to deal with various situations [142]. AI should be regarded as a valuable tool to support and strengthen clinical judgment, not as a substitute, helping oncologists tailor personalised treatment plans for mCRPC effectively.

While these problems exist universally, the implementation of AI technologies faces additional challenges in low- and middle-income countries (LMICs). In these regions, populations often lack access to basic healthcare due to financial constraints and poor infrastructure. With a significant proportion of the population residing in rural areas, the quality of healthcare provided is often mediocre to low. This is further augmented by the lack of well-developed roads for timely access to healthcare facilities. Additionally, these areas often lack access to internet facilities. While there are pertinent solutions, such as building infrastructure and installing internet devices, the costs required to achieve this impose an immense economic burden. This highlights how LMICs are bound by limited resources and finances, creating problems in the adoption of AI systems in these areas.

Training professionals, improving healthcare access and devising innovative solutions to provide AI-based systems in LMICs is essential and requires multidisciplinary, dedicated efforts. Specifically, for mCRPC, training healthcare providers on AI tools and methods to combat therapeutic resistance can significantly enhance treatment efficacy and patient outcomes in these resource-constrained settings.

## Conclusion

The review article sheds light on the emergence of AI algorithms in prostate cancer. The successful examples of AI systems in various disciplines demonstrate how these systems can revolutionise the field in terms of the speed and accuracy of diagnosis, potentially allowing prompt intervention to delay or prevent the development of mCRPC. While treatment options for mCRPC are limited, AI offers immense potential to discover novel agents and predict the therapeutic effects of drugs. AI can be leveraged not only by physicians in their routine clinical practice to decide the best possible therapeutic approach for the patient but also provides numerous opportunities to integrate genomic data for personalised treatment. With its potential to be utilised across multiple disciplines, AI holds a promising role in combating therapeutic resistance in mCRPC.

## List of abbreviations

AI, Artificial intelligence; AR, Androgen receptor; cfDNA, circulating cell-free DNA; CRPC, Castration-resistant prostate cancer; DL, Deep learning; LMICs, Low middle income countries; ML, Machine learning; NGS, Next-generation sequencing; NHS, National Health Service.

## Conflicts of interest

The authors declare no competing interests.

## Funding

The author(s) received no financial support for the research, authorship and/or publication of this article.

## Declarations: human and animal rights and informed consent

No human or animal studies were carried out by the authors for this article.

## Ethical considerations

Ethical approval was not required for this study as it is a review paper. 

## Availability of data and materials

All data generated or analysed during this study are included in this published article.

## Author contributions

ZHE, RHS and ASF contributed to the literature search and writing of the original draft, review and editing.

SRK contributed to the conceptualisation of the review and supervision of the writing process, including critically revising the work. 

## Figures and Tables

**Table 1.** Overview of novel therapies investigated in clinical trials for mCRPC

Trial	Study type	Patient population	Intervention	Primary outcomes	Secondary outcomes	References
COU-AA-301 trial	Double-blind, placebo-controlled phase 3 trial	mCRPC (*n* = 1195)	Abiraterone acetate plus prednisone (*n* = 797) versus placebo plus prednisone (*n*= 398)	Longer median overall survival in abiraterone-prednisone group compared to placebo-prednisone group (14.8 versus 10.9 months; Hazard Ratio (HR) = 0.65; p < 0.001)	Longer time to PSA progression in abiraterone-prednisone group compared to placebo-prednisone group (10.2 versus 6.6 months; p < 0.001) Longer progression-free survival in abiraterone-prednisone group compared to placebo-prednisone group (5.6 versus 3.6 months; p < 0.001) Improved PSA response rate in abiraterone-prednisone group compared to placebo-prednisone group (29% versus 6%; p < 0.001)	[68]
COU-AA-302 trial	Double-blind, placebo-controlled phase 3 trial	mCRPC (*n* = 1,088)	Abiraterone acetate plus prednisone (*n* = 546) versus placebo plus prednisone (*n* = 542)	Longer median radiographic progression-free survival in abiraterone-prednisone group compared to placebo-prednisone group (16.5 versus 8.3 months; HR = 0.53, p < 0.001) 25% decrease in the risk of death in the abiraterone-prednisone group (HR = 0.75; p = 0.01)	Decreased risk of decline (by ≥1 point) in ECOG performance-status score by 18% in abiraterone-prednisone group (HR = 0.82; p = 0.05) Longer median time to the initiation of cytotoxic chemotherapy in abiraterone-prednisone group compared to placebo-prednisone group (25.2 versus 16.8 months; HR = 0.58; p < 0.001) Delay in the time to opiate use for cancer-related pain in abiraterone-prednisone group (HR = 0.69; p < 0.001) 51% reduction in risk of median time to PSA progression in abiraterone-prednisone group (HR = 0.49; p < 0.001)	[69]
STAAR	Randomized, open-label phase 2 trial	mCRPC (*n* = 53)	AAFP plus methylprednisolone (*n* = 24) versus OAA plus prednisone (*n* = 29)	AAFP 500 mg daily is therapeutically equivalent to OAA 1,000 mg daily based on rounded-up average days 9 and 10 testosterone levels. (1.05 ng/dl [0.04], AAFP; 1.02 ng/dl [0.03], OAA; *p* = 0.4703 for LS mean difference)	A PSA-50 response was observed in>65% of patients in both groups on days 28, 56, and 84 (*p* = NS) The averaged absolute testosterone levels ≤0.1 ng/dl were achieved in 25% of AAFP-treated patients and 17% of OAA-treated patients (*p* = NS) Adverse events were experienced by fewer AAFP-treated patients versus OAA-treated patients (18 (75.0%) versus 24 (82.8%))	[70]
AFFIRM trial	Double-blind, placebo-controlled phase 3 trial	CRPC (*n* = 1199)	Enzalutamide (*n* = 800) versus Placebo (*n* = 399)	Improved median overall survival in enzalutamide group compared to placebo group (18.4 versus 13.6 months; HR = 0.63; p < 0.001)	Reduction in: Prostate-specific antigen (PSA) level (54% versus 2%, *p* < 0.001), Soft-tissue response rate (29% versus 4%, *p* < 0.001), Quality-of-life response rate (43% versus 18%, *p* < 0.001), Time to PSA progression (8.3 versus 3.0 months; HR = 0.25; *p* < 0.001), Radiographic progression-free survival (8.3 versus 2.9 months; HR = 0.40; *p* < 0.001), Time to first skeletal-related event (16.7 versus 13.3 months; HR = 0.69; *p* < 0.001)	[71]
PREVAIL study	Double-blind, placebo-controlled phase 3 trial	Chemotherapy-naïve metastatic prostate cancer (*n* = 1,717)	Enzalutamide (*n* = 872) versus Placebo (*n* = 845)	Reduction in rate of radiographic progression-free survival at 12 months in enzalutamide group versus placebo group (65% versus 14%) (81% risk reduction; HR = 0.19; *p* < 0.001) 29% reduction in the risk of death in enzalutamide group versus placebo group (HR = 0.71; *p* < 0.001)	Enzalutamide group showed an improvement in: Time until initiation of cytotoxic chemotherapy (HR = 0.35; *p* < 0.001), Time until first skeletal-related event (HR = 0.72; *p* < 0.001), Complete or partial soft-tissue response (59% versus 5%; *p* < 0.001), Time until prostate-specific antigen (PSA) progression (HR = 0.17; *p* < 0.001) Rate of decline in PSA (78% versus 3%; *p* < 0.001)	[72]
STRIVE trial	Double-blind, placebo-controlled phase 2 trial	Non-metastatic or metastatic CRPC (*n* = 396)	Enzalutamide (*n* = 198) versus Bicalutamide (*n* = 198)	Improved median progression-free survival with enzalutamide compared to bicalutamide (19.4 versus 5.7 months) and reduced risk of progression or death by 76% (HR = 0.24; *p* < 0.001)	Intervention superior in: Time to PSA progression (HR = 0.19; *p* < 0.001), Proportion of patients with a ≥ 50% PSA response (81% versus 31%; *p* < 0.001) Radiographic progression-free survival in metastatic patients (HR = 0.32; *p* < 0.001)	[73]
TERRAIN trial	Double-blind, placebo-controlled phase 2 trial	mCRPC (*n* = 375)	Enzalutamide (*n* = 184) versus Bicalutamide (*n* = 191)	Improved median progression-free survival with enzalutamide compared to bicalutamide (15.7 versus 5.8 months; HR = 0·44; *p* < 0·0001)	Longer median time to a PSA progression event in enzalutamide group versus bicalutamide group (19.4 versus 5.8 months; HR = 0·28; *p* < 0.0001)	[74]
TAX 327 study	Randomized, nonblinded phase 3 trial	Metastatic hormone-refractory prostate cancer (*n* = 1006)	Mitoxantrone plus prednisone every 3 weeks (MP) (*n* = 337) versus docetaxel administered every 3 weeks plus prednisone (D3P) (*n* = 335) versus docetaxel administered weekly plus prednisone (D1P) (*n* = 334)	Longest median survival in D3P group (19.2 months in D3P group versus 17.8 in D1P group versus 16.3 in MP group) (HR = 0.79; p < 0.004) More patients survived ≥ 3 years in the D3P and D1P arms (18.6% and 16.6%, respectively) compared with the MP arm (13.5%)	D3P showed a similar benefit compared to MP in patients with lower and higher baseline PSA levels (HRs = 0.83 and 0.73, respectively), with a median baseline PSA of 115 ng/ml. D3P had a greater benefit than MP for patients with visceral metastases, (HR = 0.87) D3P showed similar benefits compared to MP for patients with KPS ≥ 90% and KPS 80%, (HRs = 0.75 and 0.82, respectively) D3P was more effective than MP for patients without substantial pain (HR = 0.73) and with substantial pain (HR = 0.85) D3P showed greater benefit than MP for patients with worse QOL (HR = 0.66) and a moderate benefit for those with better QOL (HR = 0.92)	[75]
TROPIC trial	Randomized open-label phase 3 study	mCRPC (*n* = 775)	Mitoxantrone plus prednisone (*n* = 377) versus Cabazitaxel plus prednisone (*n* = 378)	Improved median survival in cabazitaxel group compared to mitoxantrone group (15.1 versus 12.7 months; 30% reduction in relative risk of death (HR = 0·70; *p* < 0·0001)	Improved median progression-free survival with Cabazitaxel compared to mitoxantrone (2.8 versus 1.4 months; HR = 0· 4; *p* < 0· 0001) Cabazitaxel group had a higher: Tumor Response rate (14.4% versus 4.4%; *p* = 0.0005) PSA response rate (39.2% versus 17.8%; *p* = 0.0002) Median time to tumour progression (8.8 versus 5.4 months; HR = 0· 61; *p* < 0· 0001) Median time to PSA progression (6.4 versus 3.1 months; HR = 0· 75; *p* = 0· 001) Median time to pain progression (HR = 0· 91; *p* = 0.52)	[76]
CARD study	Randomized, open-label, clinical trial	mCRPC (*n* = 225)	Cabazitaxel plus prednisone and GCSF (A) versus either abiraterone plus prednisone or enzalutamide (B)	Clinical benefit rate was greater for first-line cabazitaxel than B (80% versus 62%; *p* = 0.039)	Overall survival was not different between groups A and B (median 37.0 versus 15.5 months; HR = 0.58; p = 0.073) nor was time to progression (median 5.3 versus 2.8 months; HR = 0.87; p = 0.52)	[77]
PROSELICA study	Randomized open-label phase 3 study	mCRPC (*n* = 1,200)	Cabazitaxel 20 mg/m^2^ (C20, *n* = 598) versus Cabazitaxel 25 mg/m^2^ (C25, *n*= 602)	Improved overall survival in C25 group compared to C20 group (14.5 versus 13.4 months; HR = 1.024)	Median PFS similar in both groups (3.5 versus 2.9 months; HR = 1.099) PSA response rates significantly higher in the C25 arm (29.5% versus 42.9%; 50% decline in PSA from baseline (nominal *p* < 0.001). Median time to PSA progression was longer for C25 arm compared to C20 arm (6.8 versus 5.7 months; HR for C20 v C25 = 1.195)	[78]
FIRSTANA study	Open-label phase III study	mCRPC (*n* = 1168)	Cabazitaxel 20 mg/m^2 ^(C20) versus Cabazitaxel 25 mg/m^2^ (C25) versus Docetaxel 75 mg/m^2^ (D75), plus daily prednisone.	Similar median survival in all groups (24.5 versus 25.2 versus 24.3 months) (HR for C20 versus D75 = 1.01; p = 0.997), and (HR for C25 versus D75 = 0.97; p = 0.757)	Similar median progression-free survival in all groups (4.4 versus 5.1 versus 5.3 months) Radiographic tumor responses were higher for C25 (41.6%) versus D75 (30.9%; nominal p = 0.037) Tumor responses were higher for C25 arm (41.6%) compared with D75 (30.9%; nominal p = 0.037)	[79]
					Pain PFS was longer in D75 compared with C25 ((HR for C25 versus D75 = 1.19; nominal *p* = 0.035), and (HR for C20 versus D75 = 1.19; nominal *p* = 0.118)	
Corn *et al *[80]	Randomized, open-label, phase 1-2 study	Progressive mCRPC (*n* = 160 in phase 2)	Cabazitaxel (*n* = 79) versus Cabazitaxel plus Carboplatin (*n* = 81)	A maximum tolerated dose of cabazitaxel of 25 mg/m^2^ and carboplatin of AUC 4 was selected.	Improved median progression-free survival with combination compared to Cabazitaxel alone (7.3 versus 4.5 months; HR = 0· 9; *p* = 0· 018)	[80]
D9902B trial	Double-blind, placebo-controlled, phase 3 trial	mCRPC (*n* = 512)	Sipuleucel-T (*n* = 341) versus Placebo (*n* = 171)	Improvement in median survival in the sipuleucel-T group compared to placebo (25.8 versus 21.7 months; 22% reduction in mortality risk; HR = 0.77; *p* = 0.04)	Similar time to objective disease progression in sipuleucel-T group compared to placebo (3.7 versus 3.6 months; HR = 0.95; *p* = 0.63) PSA baseline reductions of at least 50% on two visits at least 4 weeks apart observed in sipuleucel-T group (2.6%), as compared with placebo group (1.3%).	[81]
KEYNOTE-199	Open-label, phase 2 study	mCRPC treated with docetaxel and one or more targeted endocrine therapies (*n* = 258). Cohort 1: RECIST-measurable PD-L1-positive (*n* = 133) versus Cohort 2: PD-L1-negative (*n* = 66) versus Cohort 3: bone-predominant disease, regardless of PD-L1 expression (*n* = 59).	Pembrolizumab every 3 weeks for up to 35 cycles	Objective response rate in cohort 1 was 5% as compared to 1% in cohort 2	Median overall survival was 9.5 months in cohort 1, 7.9 months in cohort 2, and 14.1 months in cohort 3 Disease control rate was 10% in cohort 1, 9% in cohort 2, and 22% in cohort 3.	[82]
Tannock *et al* [83]	Randomized trial	Symptomatic hormone-resistant prostate cancer (*n* = 161)	Mitoxantrone plus prednisone versus prednisone alone	Improved palliative response in mitoxantrone group compared to placebo (29% versus 12%; *p* = 0.01)	Response duration was longer for treatment with mitoxantrone plus prednisone than for prednisone alone (median 43 versus 18 weeks, p < 0.0001)	[83]
Kantoff *et al* [84]	Randomized trial	Hormone-refractory prostate cancer (*n* = 242)	Mitoxantrone plus hydrocortisone (M1H) versus hydrocortisone	No difference in survival between the treatment arms (median duration, 12.6 months for hydrocortisone versus 12.3 months for M1H; *p* = 0.77)	Longer median time to treatment failure and disease progression after initiation with M1H versus hydrocortisone (3.7 versus 2.3 months; *p* = 0.0254 for treatment failure; *p* = 0.0218 for disease progression)	[84]
PROfound study	Randomized, open-label, phase 3 trial	mCRPC who had disease progression while receiving a new hormonal agent (e.g., enzalutamide or abiraterone) Cohort A: patients with BRCA1/2 or ATM mutations, Cohort B: patients with a mutation in at least one of 12 other HRR genes (BARD1, BRIP1, CDK12, CHEK1, CHEK2, FANCL, PALB2, PPP2R2A, RAD51B, RAD51C, RAD51D, or RAD54L).	Olaparib versus physician’s choice of enzalutamide or abiraterone.	Longer imaging-based progression-free survival in the Olaparib group compared to the control group (7.4 versus 3.6 months; HR = 0.34; *p* < 0.001) in cohort A	Higher objective response rate among patients who could be evaluated in olaparib group (22%) compared to control group (4%) (OR = 5.93) in both cohorts Longer median overall survival in olaparib group than in control group (17.5 versus 14.3 months; HR = 0.67) in both cohorts PSA50 response confirmed in 30% in the olaparib group and 10% in the control group in both cohorts	[85]
TRITON2 trial	Open-label, phase 2 study	mCRPC with a deleterious or suspected deleterious germline or somatic BRCA1 or BRCA2 mutation, and who had disease progression after previously receiving therapy with a novel hormonal agent plus one taxane chemotherapy.	Rucaparib versus control medication	Longer duration of imaging-based progression-free survival in the rucaparib group than in the control group, both in the BRCA subgroup (median, 11.2 months versus 6.4 months, respectively; HR = 0.50; *p* < 0.001) and in the intention-to-treat group (median, 10.2 months versus 6.4 months, respectively; HR = 0.61; *p* < 0.001)	In the BRCA subgroup, the median overall survival was longer in the rucaparib group versus the control group (24.3 versus 20.8 months; HR = 0.81; *p* = 0.21) Frequency of a confirmed objective response in the rucaparib group and the control group was 45% and 17% respectively, in the BRCA subgroup; 35% and 16% respectively, in the intention-to-treat population; and no response and 14% respectively, in the ATM subgroup	[86]
TRITON3 trial	Open-label, controlled, randomized, phase 3 trial	mCRPC with a *BRCA1*, *BRCA2*, or *ATM* alteration and who had disease progression after treatment with a second-generation androgen-receptor pathway inhibitor (ARPI) (*n* = 485)	Rucaparib (*n* = 270) versus physician’s choice (docetaxel or a second-generation ARPI [abiraterone acetate or enzalutamide] (*n* = 135)	In BRCA subgroup, longer imaging-based progression-free survival in the rucaparib group compared to the control group (11.2 versus 6.4 months; HR = 0.50, *p* < 0.001) Longer median imaging-based PFS in the intention-to-treat population (10.2 months versus 6.4 months; HR = 0.61, *p* < 0.001)	Objective response rate in the BRCA subgroup and the ATM subgroup was 45% versus 17% and 0% versus 14%, respectively. The overall survival in the BRCA subgroup was 24.3 months versus 20.8 months (HR = 0.81, *p* < 0.21) 50% PSA response rate was seen in 55% of the patients with rucaparib as compared to 27% in the control group.	[87]
PROpel trial	Double-blind, phase 3 trial	mCRPC (*n* = 796)	Abiraterone plus Olaparib (*n* = 399) versus Abiraterone plus placebo (*n* = 396)	Longer imaging-based progression-free survival in intervention group as compared to placebo group (24.8 versus 16.6 months; HR = 0.66; *p* < 0.001)	Immature overall survival data (28.6% maturity; HR = 0.86)	[88]
TALAPRO-2 study	Randomized, double-blind, phase 3 trial	mCRPC receiving ongoing androgen deprivation therapy (*n* = 805)	Talazoparib plus Enzalutamide versus Placebo plus Enzalutamide	Median rPFS was not reached (95% CI 27· 5 months-not reached) for talazoparib plus enzalutamide and 21· 9 months for placebo plus enzalutamide (HR = 0.63; *p* < 0· 0001)	Immature overall survival data	[89]
MAGNITUDE trial	Phase 3, randomized, double-blinded study	mCRPC with HRR+ mutations (*n* = 423)	Niraparib + Abiraterone versus Placebo + Abiraterone	Longer radiographic progression-free survival in HRR+ group with niraparib group as compared to placebo group (16.5 versus 13.7 months; HR = 0.73; *p* = 0.022) Longer radiographic progression-free survival in BRCA1/2 group with niraparib as compared to placebo group (16.6 versus 10.9 months; HR = 0.53; *p* = 0.001)	In the HRR+ cohort, niraparib COMBINATION delayed TCC (HR = 0.59; *p* = 0.011) and TSP (HR = 0.69; *p* = 0.04), which was also observed in the BRCA1/2 subgroup Niraparib combination prolonged time to PSA progression and led to higher ORR in the HRR+ and BRCA1/2 groups (time to PSA progression and rPFS were strongly correlated, with an overall *r* = 0.67 (95% CI, 0.56-0.75))	[90]
VISION trial	Open-label, phase 3 trial	mCRPC patients previously treated with at least one ARPI and one or two taxane regimens and who had PSMA-positive gallium-68 (^68^Ga)-labeled PSMA-11 positron-emission tomographic-computed tomographic scans	^177^Lu-PSMA-617 plus protocol-permitted standard care (*n* = 551) versus standard care alone (n = 280)	Longer imaging-based progression-free survival (8.7 versus 3.4 months; 60% improvement; HR = 0.40; *p* < 0.001) and overall survival (15.3 versus 11.3 months; 38% improvement; HR = 0.62; *p* < 0.001) in intervention group compared to placebo	Intervention superior in: Median OS (15.3 versus 11.3 months; HR = 0.62; *p* < 0.001), Median time to HRQOL (14.3 versus 2.9 months; HR = 0.45; *p* < 0.001); Median time to pain worsening (1.0 versus 0.5 months; HR = 0.65; *p* < 0.001)	[91]
ALSYMPCA trial	Randomized, double-blind, placebo-controlled study	Progressive mCRPC (*n* = 921)	Radium-223 (*n* = 614) versus Placebo (*n* = 307)	Improved median overall survival in radium-223 group compared to placebo (14.0 versus 11.2 months; 30% reduction in the risk of death HR = 0.70; *p* = 0.002)	Intervention prolonged the: Time to the first symptomatic skeletal event (median, 15.6 months versus 9.8 months; HR = 0.66; *p* < 0.001 Time to an increase in the total alkaline phosphatase level (HR = 0.17; *p* < 0.001) Time to an increase in the PSA level (HR = 0.77; *p* < 0.001)	[92]
HR = Hazard Ratio; AAFP = Abiraterone acetate fine particle; OAA = Originator abiraterone acetate; MP = Mitoxantrone-prednisone every 3 weeks MP; D3P = Docetaxel administered every 3 weeks; D1P = Docetaxel administered weekly plus prednisone; rPFS = radiographic progression free survival; C20 = Cabazitaxel 20 mg/m^2^; C25 Cabazitaxel 25 mg/m^2^; D75 = Docetaxel 75 mg/m^2^						

**Table 2.** Current literature available on AI applications in diagnosis, grading and treatment of prostate cancer.

Study	Study design	Primary outcome	Secondary outcome	Results	References
**Pathomics**					
Morozov *et al* [93]	Systematic review & meta-analysis	AI accuracy in differentiating between prostate cancer and benign hyperplasia	AI accuracy in determining Gleason grade and agreement among AI and pathologists	The sensitivity for diagnosing prostate cancer was over 90%, with a range of 87%-100%, while the specificity varied between 68% and 99%.	[93]
PANDA challenge	Prospective	Development of reproducible AI algorithms for Gleason grading using digitized prostate biopsies	Validation of AI algorithms' performance on independent cross-continental cohorts	Agreements with expert uropathologists on United States and European validation sets: 0.862 (κ, 95% CI, 0.840-0.884) and 0.868 (95% CI, 0.835-0.900).	[96]
DeepDx	Prospective Cohort	Performance validation of DeepDx for prostate cancer diagnosis and grading using an independent external dataset	Evaluation of DeepDx’s value to the general pathologist	DeepDx achieved high accuracy for prostate cancer detection similar to original pathology reports and higher concordance	[97]
Paige prostate	Prospective Cohort	Diagnostic performance of pathologists diagnosing prostatic core needle biopsies unaided and with AI assistance	Not applicable	Reduction in the number of atypical small acinar proliferation reports, immunohistochemistry studies, second opinions, and time required for reading and reporting slides	[98]
Radiomics					
Wang *et al* [99]	Prospective observational	Prediction of BM in prostate cancer using texture features from mp-MRI	Comparison of predictive performance with PSA level and Gleason Score	Texture features from T2-w and DCE T1-w MRI showed strong association with BM (*p* < 0.01)	[99]
Li *et al* [100]	Prospective observational	Development of AI model using TRUS images to predict prostate cancer	Comparison of AI model diagnostic performance with radiologists and clinical models	Better diagnostic efficacy than senior radiologists (AUC: 0.667). Detected 82.9% of prostate cancer cases versus 55.8% by radiologists	[100]
Faiella *et al* [101]	Systematic review	Evaluation of AI models for LNI detection and prediction in prostate cancer	Comparison of AI model performance with standard nomograms and imaging modalities	MRI-based AI models showed comparable LNI prediction accuracy to standard modalities	[101]
Genomics					
Decipher, Prolaris, Oncotype Dx Study	Systematic review	Evaluation of Decipher, Prolaris, and Oncotype Dx for prognostication in localized prostate cancer	Assessment of their impact on treatment decisions and patient outcomes	Decipher, Prolaris, Oncotype Dx demonstrated rigorous quality criteria and potential clinical utility in prognostication of localized prostate cancer providing additional prognostic information beyond clinicopathologic variables	[102]
Dadhania *et al* [103]	Prospective cohort	Development of a DL algorithm to identify ERG rearrangement status in prostate cancer based on digitized slides	Not applicable	All models showed similar ROC curves with AUC results ranging between 0.82 and 0.85. Sensitivity and specificity of the 20×- model was 75.0% and 83.1%, respectively	[103]
Mena *et al* [104]	Prospective cohort	Development of a classifier to predict the occurrence of prostate cancer using gene expression data and providing understandable explanations to assist pathologists	Identification of relevant genes for prostate cancer screening	RF algorithm with majority class down sampling achieved an average sensitivity of 0.90, specificity of 0.8, and an AUC of 0.84. Relevant genes include DLX1, MYL9, FGFR, CAV2, and MYLK	[104]
Recurrence and biomarker					
Huang *et al* [105]	Prospective cohort	Development of an AI-powered method for predicting 3-year biochemical recurrence of prostate cancer	Identification of a new potential prostate cancer biomarker, TMEM173, related to the STING pathway	The AI model achieved an AUC of 0.78 for predicting 3-year biochemical recurrence, outperforming Gleason Grade Group (AUC = 0.62).	[105]
Lui *et al* [106]	Systematic review	Accuracy of AI-based models in predicting biochemical recurrence (BCR) of prostate cancer post-prostatectomy	Comparison of AI models with traditional BCR prediction methods; Impact of radiological features on AI performance	AI demonstrated high accuracy, especially when incorporating radiological features, occasionally outperforming traditional prediction methods. However, due to limited high-quality studies and insufficient external validation, further research is needed to confirm the reliability and clinical applicability of AI-based techniques.	[106]
Eminaga *et al* [107]	Observational study	AI-based system for predicting recurrence and mortality in prostate cancer using histology images	Comparison of AI model with existing grading systems; Agreement among pathology experts	AI-based prediction system outperformed existing grading systems and demonstrated superiority in categorizing PCa into four distinct risk groups. High consensus was observed among pathology experts. AI may aid in informed clinical decision-making for PCa patients.	[107]
Research and drug discovery					
CancerOmicsNet	Prospective observational	Development of CancerOmicsNet to predict the therapeutic effects of kinase inhibitors across various tumors using a graph neural network	NA	CancerOmicsNet achieved an AUC of 0.83 in predicting therapeutic effects, outperforming other approaches	[108]
AndroPred	Prospective observational	Development of AI algorithms to predict AR inhibitors using a dataset of 2242 compounds	Validation of predictive models through experimental assays	The DL-based prediction model outperformed others with accuracies of 92.18% and 93.05% on the training and test datasets, respectively	[109]
Registry					
CAPRI-3	Retrospective observational	Demonstrating the reliability and efficiency of AI-driven patient identification and data collection for metastatic prostate cancer registry	Not applicable	Completeness and accuracy of automated data extraction were 92.3% or higher, except for date fields and inaccessible data	[110]
AUC = Area under curve; ROC = receiver operating characteristic					

**Table 3.** Ongoing trials incorporating artificial intelligence to develop robust algorithms in the management of prostate cancer.

Trial	Patient population	Intervention	Objective	References
Prospective validation of pathology-based artificial intelligence diagnostic model for lymph node metastasis in prostate cancer	Patients with prostate cancer undergoing radical prostatectomy and pelvic lymph node dissection	AI-based diagnostic model analyzing the whole-slide images	Assess the diagnostic accuracy and clinical utility of a pre-existing AI system for prostate cancer lymph node metastasis detection	[111]
AI based measurements of tumor burden in PSMA PET-CT	Patients referred to a clinically indicated 18F-PSMA-1007 PET-CT scan at Skåne University Hospital, Lund or Malmö, Sweden	AI-based detection and quantification of suspected tumour/metastases in PSMA PET/CT scans	Evaluate how the total tumor burden (cm^3^) predicts overall survival	[112]
Imperial Prostate 6 - Cancer Histology Artificial Intelligence Reliability Study. (IP6-CHAIROS)	Adults with a prostate (either cis-male gender or trans-female gender with no prior hormone use at all) undergoing prostate biopsy because of an elevated serum PSA or abnormal digital rectal exam, who have undergone a pre-biopsy mp-MRI and advised to undergo prostate biopsies	Biopsy & imaging	To evaluate the diagnostic accuracy and health economic value of the Galen Prostate AI system for triaging pathology slides within the NHS context.	[113]
Artificial Intelligence and Radiologists at Prostate Cancer Detection in MRI: The PI-CAI Challenge	Men suspected of harboring clinically significant prostate cancer (csPCa) with elevated PSA levels (≥ 3 ng/ml) or abnormal digital rectal exam findings. Patients must not have a history of prior prostate treatment or positive histopathology findings (ISUP ≥ 2)	Histopathology and MRI	Validate the diagnostic performance of AI algorithms and radiologists in detecting and diagnosing csPCa in MRI, comparing their efficacy and identifying the optimal AI model and the effects of imaging techniques and reader experience on diagnostic accuracy	[114]
Accelerated Body Diffusion-Weighted MRI Using Artificial Intelligence (CeleScan-R)	Cancer patients aged 18 and older who have undergone one of the following MRI examinations: whole-body for multiple myeloma, metastatic prostate cancer, metastatic breast cancer; stacked abdomen/pelvis for liver metastases, pancreatic cancer, gynecological cancers, gastrointestinal cancers; multiparametric prostate exam for primary prostate cancers	Whole-body diffusion-weighted MRI (WBDWI) using quickDWI, an accelerated technique with DL denoising filters, reducing acquisition times by up to 50%	To evaluate the clinical quality of quickDWI images compared to conventional MRI, aiming to facilitate wider adoption, reduce costs, and improve patient experience	[115, 116]
